# Commonalities and distinctions of pediatric patient and family engagement in clinical care, education, and research contexts: Protocol for a scoping review

**DOI:** 10.1371/journal.pone.0330104

**Published:** 2025-08-08

**Authors:** Brooke Allemang, Francine Buchanan, Pranshu Maini, Dalya Kablawi, Lin Li, Linda Nguyen, Kimberly Courtney, Jessie Cunningham, Carla P. Southward, Kristin Cleverley, Sarah Munce, Alene Toulany

**Affiliations:** 1 Office of Patient, Family, and Community Engagement, The Hospital for Sick Children, Toronto, Ontario, Canada; 2 Child Health Evaluative Sciences, SickKids Research Institute, Toronto, Ontario, Canada; 3 Factor-Inwentash Faculty of Social Work, University of Toronto, Toronto, Ontario, Canada; 4 Institute of Health Policy, Management and Evaluation, University of Toronto, Toronto, Ontario, Canada; 5 Faculty of Health Sciences, McMaster University, Hamilton, Ontario, Canada; 6 International Association for Public Participation Canada, Ottawa, Ontario, Canada; 7 Lawrence S Bloomberg Faculty of Nursing, University of Toronto, Toronto, Ontario, Canada; 8 McCain Centre for Child, Youth & Family Mental Health and Azrieli Adult Neurodevelopmental Centre, Centre for Addiction and Mental Health, Toronto, Ontario, Canada; 9 Faculty of Social Work, University of Calgary, Calgary, Alberta, Canada; 10 CHEO Research Institute, Ottawa, Ontario, Canada; 11 Ontario Child Health SUPPORT Unit, Ottawa, Ontario, Canada; 12 Health Sciences Library, The Hospital for Sick Children, Toronto, Ontario, Canada; 13 Bloorview Research Institute, Holland Bloorview Kids Rehabilitation Hospital, Toronto, Ontario, Canada; 14 Faculty of Medicine University of Toronto, Toronto, Ontario, Canada; 15 Campbell Family Mental Health Research Institute, Centre for Addiction and Mental Health, Toronto, Ontario, Canada; 16 Rehabilitation Sciences Institute, University of Toronto, Toronto, Ontario, Canada; 17 Division of Adolescent Medicine, The Hospital for Sick Children, Toronto, Ontario, Canada; 18 Institute of Clinical Evaluation Sciences (ICES), Toronto, Ontario, Canada; Xiamen University - Malaysia Campus: Xiamen University - Malaysia, MALAYSIA

## Abstract

**Background:**

Pediatric patient and family engagement is an active and collaborative process, that involves children, adolescents, and family members with lived experience contributing to the design, implementation, and evaluation of healthcare services. Prior studies have highlighted the patient engagement methods and impact in clinical care, education, and research. However, gaps remain in understanding the commonalities and distinctions of engagement approaches, patient/family partner roles, and outcomes in clinical care, education, and research contexts. Further, research examining the nuances of pediatric patient and family engagement within healthcare delivery, education, and research in pediatric institutions is needed to streamline efforts.

**Objective:**

This scoping review will identify the commonalities of and distinctions between pediatric patient and family engagement in clinical care, education, and research contexts in pediatric healthcare institutions.

**Methods:**

A scoping review, conducted in collaboration with a team of adolescent, young adult, and family partners, will allow us to systematically map out key concepts, evidence, and knowledge gaps regarding pediatric patient and family engagement in clinical care, education, and research. We will follow the Joanna Briggs Institute framework in the design and conduct of the review and guidance on engaging knowledge users within scoping reviews. The protocol for this scoping review has been registered with the Open Science Framework database (https://osf.io/63qx5).

**Results:**

This study will describe the engagement types, approaches, and outcomes of pediatric patient and family engagement employed within clinical care, education, and research settings, highlighting commonalities and distinctions across contexts. In doing so, it will identify potential opportunities for collaboration and resource-sharing based on the context of engagement and provide needed clarity on streamlining pediatric patient and family engagement approaches within pediatric institutional settings.

**Conclusions:**

It is anticipated that the results will produce preliminary evidence of relevance to pediatric institutions seeking to consolidate engagement practices across clinical care, education, and research domains.

## Introduction

Pediatric patient and family engagement is an active and collaborative process, that involves children, adolescents, and family members with lived experience meaningfully contributing to the design and implementation of healthcare services, educational initiatives, and research studies [1]. Guided by the aim of improving healthcare systems, pediatric patient and family engagement is vital for developing and enhancing healthcare systems by ensuring interventions and studies align with patient priorities [1]. Patient engagement in health research has been operationalized across pediatric and adult settings, and the benefits of such approaches are well-documented, including enhanced relevance of study findings, more inclusive recruitment, and results that can be more readily implemented into clinical practice [2]. The incorporation of children and adolescents’ experiences, knowledge, and perspectives into healthcare design and research can improve service delivery and the uptake of evidence into clinical practice [2]. Engaging adolescents in decision-making, program design, and implementation can help ensure services are culturally relevant, equitable and aligned with their preferences [3,4]. Further, there is evidence that actively involving pediatric patients in clinical care, education, and research results in positive outcomes for the healthcare system (e.g., better treatment outcomes), research (e.g., improved uptake of findings), healthcare providers (e.g., better understanding of patient needs), and children and adolescents themselves (e.g., improved self-confidence, development of skills) [5–8].

Systematic and scoping reviews have outlined strategies for patient engagement in different domains, including clinical care, quality improvement, and research [9,10]. For instance, a systematic review of 48 studies identified strategies and contextual factors that enable patient engagement in the design, delivery, and evaluation of healthcare services [9]. Strategies ranged from consultative unidirectional feedback to patient partnership and co-design, and resulting outputs varied based on the level of engagement employed [9]. Methods for engaging patients in research are typically guided by frameworks, including Arnstein’s ladder of citizen participation [11], the Canadian Institutes of Health Research (CIHR)’s patient engagement framework [1], or the McCain model of youth engagement [12]. Recent studies have identified principles (e.g., trust, transparency), foundational components (e.g., resources, evaluation), contexts (e.g., attitudes, expectations), actions (e.g., methods, outputs), and outcomes (e.g., research uptake) of engaging patients in research [10,13,14]. In addition, community-based research approaches have been employed to develop health promotion and educational interventions with knowledge users that can be applied in healthcare settings [15,16]. Despite the existence of these frameworks, principles, and approaches, there is a lack of consensus as to the optimal methods and practices that support patient and family engagement across sectors to enable sustainable partnerships, as opposed to time-limited, siloed engagement opportunities [17]. Although methods of co-production in healthcare have been applied to and described in the areas of clinical care, research, quality improvement, and medical education [18], gaps remain in understanding the commonalities of and distinctions between engagement in clinical care, education, and research contexts. In fact, Boivin et al. recently called for an ecological synthesis of the literature to understand the environments in which engagement occurs to produce comprehensive policies that attend to organizational context [17].

There are unique developmental considerations involved in partnering with children, adolescents, and their families in health systems improvement and research [7,19,20]. Such considerations include incorporating appropriate protections for adolescents (e.g., allies to mitigate power differentials), establishing peer-to-peer relationship building and mentorship, offering flexible engagement opportunities conducive to adolescents’ ages, developmental stages, and preferences, and negotiating evolving caregiver roles, as appropriate [12,19,21]. However, much of the literature has focused on methods, frameworks, and practices for engaging adult patients in healthcare. While health-related youth advisory councils are widespread [22–24], and recommendations for effective youth engagement in research have been developed [19], little attention has been paid to the institutional factors within pediatric settings that enable or hinder engagement across the continuum from research to service delivery. Further, the literature has not addressed how engagement approaches within pediatric institutions (i.e., pediatric organizations providing clinical care, delivering health education/health promotion activities, or conducting health research/quality improvement initiatives) differ based on the engagement context and resources available. Research examining the nuances of pediatric patient and family engagement between clinical care, education, and research contexts is needed to streamline efforts and illuminate the infrastructure required to optimize engagement practices across these domains at an institutional level.

### Research objective

The proposed scoping review will map out the published and unpublished (grey) literature on pediatric patient and family engagement in clinical care, education, and research. We will address the following research question: what are the commonalities of and distinctions between pediatric patient and family engagement approaches when applied in clinical care, education, and research contexts in pediatric institutions?

## Methods

A scoping review, conducted in collaboration with a team of adolescent and young adult patient partners and family partners, will permit systematically mapping key concepts, evidence, and knowledge gaps regarding pediatric patient and family engagement in clinical care, education, and research [25,26]. This methodology is appropriate given the aim of assessing and understanding the breadth and depth of knowledge in this area [25,26]. The protocol for this scoping review has been registered with the Open Science Framework database (https://osf.io/63qx5). We will follow the Joanna Briggs Institute (JBI) framework [27] in the design and conduct of the review and guidance on engaging knowledge users within scoping reviews [28]. We will adhere to the Preferred Reporting Items for Systematic Reviews and Meta-Analysis Extension for Scoping Reviews (PRISMA-ScR) [29] in the reporting of findings.

### Patient engagement strategy

An integrated knowledge translation approach will be adopted in this scoping review, whereby research is co-produced with individuals with lived experience [30]. Guided by the principles of patient-oriented research (POR), including mutual respect, support, inclusiveness, and co-building [1], this scoping review will be conducted in collaboration with a team of patient and family partners to bring the perspectives of those with lived experience to this review. While patient engagement has not traditionally been part of the scoping review methodology, there is a growing interest in and reported benefits of including patient and family perspectives in the conduct of literature reviews [28]. Given the focus of this scoping review, an integrated knowledge translation approach is fitting to ensure the research reflects and responds to the needs, priorities, and experiences of patients and families.

We anticipate recruiting four research partners in total; two adolescents and young adults (aged 15–30) and two family members (e.g., parents, caregivers, siblings, extended family members) with experiences of engagement to act as partners in the conduct of our research. This group size will allow for meaningful and active partnership with a small group of committed individuals from diverse backgrounds in terms of gender, lived experience, and race/ethnicity. Eligibility criteria for patient and family partners will include: i) being a patient or family member of a patient who has received care at a pediatric hospital in Canada, ii) having an interest in sharing their perspectives on pediatric patient and family engagement, and iii) possessing experience being engaged as an advisor or partner in prior research. Partners will be recruited through pre-existing relationships with the research team given the nature of this opportunity and the eligibility criteria. Detailed information letters about the opportunity will be emailed to select individuals with prior experience and/or an expressed commitment to pediatric patient and family engagement by the research team. Those with an interest in learning more will be invited to contact the research team to schedule a virtual interview. Interviews will provide prospective partners with information about the time commitment, potential roles, compensation structure, and to learn about the hopes, strengths, and interests of partners. Prior to onboarding partners, roles will be clearly defined and communicated, partners will have opportunities to inquire about the project, and barriers to participation will be feasibly addressed. Those meeting criteria and who can commit to the project timeline will be formally onboarded. While the research team recognizes the perspectives of four partners cannot represent the broader needs and perspectives of an entire population, efforts will be made to purposefully select individuals with differing lived experiences (e.g., a variety of health conditions), identities (e.g., patients versus family members, genders), and views to provide rich insights into the topic under investigation.

Ethical guidelines for the involvement of adolescent, young adult, and family partners in research are lacking, despite evidence that this is a critical area to explore [31,32]. Research ethics board approval for the involvement of patient and family partners in research is not required at the host institution, given they are not study participants. Further, consent from patient and family partners will not be obtained as they will be members of the research team, hired for their expertise. Their consent for becoming involved in the research process will be implied by their application to the patient and family partner role. Though oversight of the patient and family partners by the research ethics board will not be provided, the research team will employ developmentally appropriate and ethical considerations for involving people with lived experience in research [31]. These will include co-development of brave space guidelines, flexible meeting times, sharing decision-making power within the partnership, using lay language to make materials accessible and developmentally appropriate, offering partners choice in how they contribute to the review, and clear documentation of how partner feedback is being implemented throughout the review. A youth engagement specialist focused on adolescent and young adult engagement in research and clinical care at the host institution will also be available to support the partners and team, should the need arise. In addition, guidelines will be employed to ensure that any personal experiences shared by partners during meetings will be kept confidential and will not be shared outside of meetings without permission [31].

Partnership procedures will be co-developed based on the CIHR patient engagement framework [1]. We anticipate holding virtual meetings with the patient and family partners on a bi-weekly basis during data screening, extraction, and interpretation over a four-month period (May – September 2025). Patient and family partners will be compensated for their time in alignment with CIHR’s Patient Partner Compensation Guidelines [33] for meeting attendance (including preparation) and asynchronous activities. A terms of reference document that we have co-developed with knowledge users on another project [34], which includes values and principles, guidance on defining roles, responsibilities, and opportunities, decision-making processes, and how knowledge users will be recognized for their contributions, will help to guide our partnership activities in the scoping review. Patient and family partners will be invited to contribute their perspectives and additions to this living document to ensure it is reflective of their hopes for involvement. Communication channels will be established with patient and family partners and may include the development of shared drives for housing review materials and/or email threads. Efforts to sustain the engagement of patient and family partners over the four-month period will include mutually agreed upon meeting times, co-developed meeting agendas, options for synchronous and asynchronous participation, and developing roles/responsibilities based on partner preferences.

The patient and family partners will collaborate in the creation of the knowledge generated through this scoping review; as such, dissemination outputs will be co-developed with messages and methods of delivery tailored according to the needs of knowledge users [35,36]. They will assist with developing the data extraction tool, advise on study relevance, review preliminary results, provide their feedback on the interpretation of findings, contribute to manuscript preparation, and participate in knowledge mobilization activities, including co-presentations at conferences and community events. All partners will be included as co-authors on the scoping review results article to reflect their essential contributions. Appropriate training will be provided to patient and family partners by the study team to support their active involvement in the scoping review. Methods of delivery for training will be developed based on the preferences of patient and family partners and may include a variety of options tailored to their needs (e.g., virtual education sessions, using videos, through small group work). Insights and reflections from patient and family partners regarding the scoping review will be obtained through group discussions at bi-monthly virtual meetings. While high-level meeting minutes will be taken to capture team-based decisions about concepts within the review, confidentiality will be discussed during onboarding sessions to introduce partners to this review, and each partner will have autonomy in determining what they would like to share. No identifying information about specific partners’ own experiences will be collected or published in the final review.

### Search strategy

A systematic search strategy has been developed with the support of an experienced research librarian at the host institution to identify relevant published literature in electronic databases (e.g., MEDLINE, EMBASE, CINAHL, PsycINFO; S1 Appendix). The search string was established in alignment with study inclusion criteria, guided by the “PCC” mnemonic (population, concept, context) [26] and compared against Medical Subject Headings to ensure relevance (see S1 Appendix). Given our focus on pediatric patient and family engagement, our population of interest for the phenomena under study is pediatric patients (aged 4–18 years based on prior studies [7]) and the age at which transfer out of pediatric care typically occurs [37] and/or family members (e.g., parents, caregivers, siblings, extended family members) carried out within pediatric institutions. Mean age of the patient population will be used, when necessary. The concept to be explored is engagement, including what engagement approaches (e.g., priority setting activities, document reviews, co-design) are described. The context is pediatric institutions or organizations providing clinical care, delivering health education/health promotion activities, or conducting health research/quality improvement initiatives (e.g., hospitals, primary care clinics, research centres, community-based organizations) in any geographic location. We will include quality improvement projects, qualitative, quantitative, and mixed methods studies published in English (for feasibility purposes) between 2011 (when Canada’s Strategy for Patient-Oriented Research was established [38]) and present. Conference abstracts, dissertations, reviews, and protocols will be excluded. Reference lists of relevant articles (including systematic or scoping reviews) will also be searched. To date, we have completed the initial search of the published literature and identified relevant articles but have not yet scanned the reference lists of articles. A targeted search of grey literature will be also conducted by our team to identify frameworks and guidance documents from funding bodies, community organizations, and other institutions relevant to our research question given the importance of these data sources. We will search for grey literature in databases like OpenGrey, Grey Matters, and Grey Literature Report. The grey literature search has not yet been conducted.

### Evidence screening and selection

Articles and documents will be imported into Covidence for screening and extraction. Duplicates will be removed prior to level 1 screening. Titles and abstracts will be screened by two independent reviewers based on the research objective and inclusion criteria. Pilot testing at level 1 screening will be conducted to ensure consistency across reviewers. A sample of approximately 50 articles will be reviewed by study team members, including patient and family partners, and inclusion criteria may be adjusted if there is < 70% agreement on decisions to include/exclude articles and documents [39,40]. Level 1 screening of the published literature has been completed.

Next, full-text versions of potentially relevant literature and documents will be reviewed by two independent reviewers and compared against study inclusion criteria. Following a similar process to level 1, pilot testing will be conducted at level 2 on approximately 25% of the articles to assess inter-rater reliability [39,41,42]. Any disagreements that arise in the screening process will be resolved by consensus. Study selection processes, including reasons for exclusion at level 2, will be described and presented in a flow diagram. Level 2 screening was completed in June 2025.

### Data extraction

A data extraction tool will be developed in collaboration with our patient and family partners to categorize our findings according to the JBI guidelines [27]. Examples of characteristics to be extracted are as follows: publication year, country of publication, publication type, patient/family partner characteristics (e.g., age, gender, lived experience for which their expertise was sought, sociodemographic information, illness/diagnosis), engagement context (clinical care [including quality improvement], education, research), type of pediatric institution (e.g., hospital, research centre, community organization), engagement approach, level/degree of engagement, roles of patient/family partners (e.g., consultant, advisory council member, co-researcher), stage(s) at which engagement occurred, outcome(s) of engagement (e.g., improved skills, increased recruitment, greater relevance), impact of research results, and training completed (e.g., for patient and family partners, clinicians, or researchers). The tool will be pilot tested with two reviewers on five studies to ensure it captures all relevant data and modified as needed. Data extraction will be completed by a primary reviewer and verified by a second reviewer. Characteristics to be collected may evolve as literature is reviewed and in discussion with our patient and family partners. Any additions/modifications to the data extraction tool will be documented for transparency. We anticipate completing data extraction by September 2025.

### Data analysis

Quantitative data will be summarized using percentages, means or medians. Qualitative content analysis [43] will be used to count and compare content from various publication types (e.g., the number of qualitative studies on pediatric patient engagement in research). Content analysis [43] will be used to analyze and present the engagement approaches, roles of patient/family partners, and outcomes according to context (e.g., clinical care, education, research). One team member will code the extracted data, engage in discussions about the findings with the broader team, and review emergent patterns. Preliminary findings will be shared with patient and family partners for feedback, group discussion, and collective interpretation. The findings about engagement within clinical care, education, and research contexts will be jointly reflected on by the team and interpreted in light of our diverse experiences and backgrounds. We will consider the implications of our learnings about commonalities and distinctions between engagement in different settings on possibilities for streamlining engagement approaches at an institutional level. A comprehensive summary will be collaboratively developed with patient and family partners based on the findings and our reflections, evidence gaps will be identified, and priorities for future research will be outlined. We expect that final results will be available in November 2025.

### Ethical considerations

Ethical approval will not be required for this scoping review as it does not involve human subjects or primary data collection.

## Discussion

While methods of engagement have been identified in the siloed contexts of clinical care, research, quality improvement, and education, this scoping review aims to support a holistic and integrated approach to engagement across sectors by exploring commonalities and distinctions. Its findings will identify what approaches, levels, and outcomes of pediatric patient and family engagement in clinical care, education, and research are reported in the literature. This study is needed as literature to date has not ascertained and mapped these concepts in the areas of clinical care, education, and research in pediatric institutions, specifically. While prior reviews have outlined engagement strategies employed in clinical care, quality improvement, health professions education or health research [9,10,44,45], a broader scan of the literature with a focus on pediatric institutions is crucial. A comprehensive view of the engagement landscape that attends to the context of engagement, while exploring avenues for bridging the areas of theory and practice, is an important next step in the field of patient and family engagement [17]. Determining engagement approaches and levels based on the setting and context of the engagement initiative will highlight potential opportunities for collaboration and resource-sharing between clinical, education, and research spheres, as well as knowledge gaps, and areas for future research. Streamlining engagement within healthcare institutions has the potential to reduce the gap between research and its implementation, expediting improvements in clinical outcomes for patients and families. In addition, breaking down silos will lead to a more coordinated healthcare experience, facilitating feedback loops where clinical challenges inform research priorities, and research findings improve clinical care. It is anticipated that the results will produce preliminary evidence of relevance to pediatric institutions, particularly those in which engagement occurs in different contexts and for different reasons. The scoping review will provide needed clarity on integrating pediatric patient engagement approaches within an institutional setting and developing strategies that apply across domains, from inclusive recruitment practices and equitable compensation strategies to guidance on reporting the outcomes and impact of engagement.

### Limitations

The proposed scoping review will be subject to some limitations. It is possible there will be more pediatric patient and family engagement literature focused on certain contexts (e.g., research) over others (e.g., health education), lending to more complete summaries of engagement approaches in these areas. We will use frequency counts and content analysis to outline the number of studies specific to each context and highlight our limitations and future directions when reporting the findings. The articles we report on will likely be disproportionately led by researchers, rather than patients or families, thus may not accurately reflect how patients and families perceive the impact of the engagement practices employed. The involvement of patient and family partners in developing the data extraction tool and interpretating the data will, therefore, be crucial. Our grey literature search will be targeted for feasibility purposes, as it is beyond the project’s scope to replicate the search strategy to the same degree as in the published literature. We recognize this could result in missing potentially relevant materials consistent with our study objectives due to publication bias [46]. Future research could aim to more rigorously examine the grey literature regarding how pediatric patient and family engagement approaches are applied within clinical care, education, and research contexts in the unpublished literature. In addition, capturing the breadth of literature relevant to our objective may prove challenging given the various terms used to describe patient and family engagement (e.g., patient and public involvement, patient engagement, service user involvement). To address this challenge, we have worked with a research librarian experienced with knowledge synthesis methodologies and in the area of patient and family engagement and will collaborate with our patient and family partners to clearly define our inclusion criteria and ensure the search strategy encompasses the range of terminology present in the literature. This review will be limited to articles published in English given a lack of resources for personnel to screen and extract these additional articles. Thus, the data extracted and findings developed through the review may not adequately reflect the experiences of marginalized populations related to engagement. We recognize the importance of examining articles and studies published in languages other than English and will consider this in future funding applications and research endeavours. Lastly, we acknowledge that the involvement of a small group of patient and family partners will not entirely reflect the breadth of perspectives held by adolescents, young adults, and family members regarding pediatric patient and family engagement. However, this scoping review will identify gaps or underrepresented perspectives in the literature and highlight implications for improving diversity in pediatric patient and family engagement.

### Strengths

Strengths of this review include the involvement of an interdisciplinary team of researchers, patient partners, and family partners who will bring intersectional and equity-informed perspectives to data extraction, analysis, and interpretation. Several members hold dual identities as researchers and individuals with lived experience, each contributing their knowledge and views to the topic under study. In addition, this team is comprised of those with an array of research experiences, from early career to senior researchers, and with a diversity of experiences in engagement. At least two team members will be involved in level 1 and 2 screening and steps will be taken to ensure inter-rater reliability. Finally, reference lists of relevant articles and existing systematic/scoping reviews will be hand-searched for additional studies that meet inclusion criteria to support a comprehensive search strategy.

### Dissemination

We will disseminate the findings of this review by publishing in an open-access peer-reviewed journal and sharing results and implications at conferences, webinars, and relevant community events, including patient and family advisory council meetings at pediatric institutions across Canada. Possible venues for dissemination via co-presentations with patient and family partners include the Ontario Strategy for Patient-Oriented Research SUPPORT Unit Research Day, the Pride in Patient Engagement in Research (PiPER) Research Day, Children’s Healthcare Canada’s Annual Conference, Kids Brain Health Network’s Annual Conference, the Patient-Centered Outcomes Research Institute Annual Meeting, the AcademyHealth Annual Conference on the Science of Dissemination and Implementation, or the International Association for Public Participation North American Conference. We will develop plain language summaries and infographics with patient and family partners to share relevant findings with knowledge users and identify appropriate channels for reaching adolescents, families, and healthcare providers, specifically. We also anticipate incorporating learnings from this review into training programs for staff wishing to engage patients and families at the host institution.

## Supporting information

S1 Appendix Literature search strategy. (DOCX)

S1 Checklist PRISMA-P 2015 Checklist.
(DOCX)

## References

[1] Canadian Institutes of Health Research. Patient Engagement Framework. https://cihr-irsc.gc.ca/e/48413.html. 2019. 2024 February 16.

[2] Birnie KA, Dib K, Ouellette C, Dib MA, Nelson K, Pahtayken D, et al. Partnering for pain: a priority setting partnership to identify patient-oriented research priorities for pediatric chronic pain in Canada. CMAJO. 2019;7:E654–E664. 10.9778/cmajo.20190060 PMC6839970 31699686

[3] Augsberger A, Young A, Toraif N, Morris M, Barnett KG. Youth engagement to achieve health equity: Are healthcare organizations and leaders prepared? Am J Community Psychol. 2023;71:410–22. 36661430 10.1002/ajcp.12656

[4] Sprague Martinez LS, Tang Yan C, Augsberger A, Ndulue UJ, Libsch EA, Pierre JS, et al. Changing The Face Of Health Care Delivery: The Importance Of Youth Participation. Health Aff (Millwood). 2020;39(10):1776–82. doi: 10.1377/hlthaff.2020.00728 33017230

[5] Jeremic V, Sénécal K, Borry P, Chokoshvili D, Vears DF. Participation of Children in Medical Decision-Making: Challenges and Potential Solutions. J Bioeth Inq. 2016;13(4):525–34. doi: 10.1007/s11673-016-9747-8 27654498

[6] Domecq JP, Prutsky G, Elraiyah T, Wang Z, Nabhan M, Shippee N, et al. Patient engagement in research: a systematic review. BMC Health Serv Res. 2014;14:89. doi: 10.1186/1472-6963-14-89 24568690 PMC3938901

[7] Teela L, Verhagen LE, van Oers HA, Kramer EEW, Daams JG, Gruppen MP, et al. Pediatric patient engagement in clinical care, research and intervention development: a scoping review. J Patient Rep Outcomes. 2023;7(1):32. doi: 10.1186/s41687-023-00566-y 36988738 PMC10060502

[8] Allemang B, Patton M, Greer K, Pintson K, Farias M, Schofield K, et al. Development of the Strengths, Skills, and Goals Matrix: a tool for facilitating strengths-based adolescent and young adult engagement in research. Res Involv Engagem. 2023;9(1):89. doi: 10.1186/s40900-023-00502-w 37794455 PMC10548729

[9] Bombard Y, Baker GR, Orlando E, Fancott C, Bhatia P, Casalino S, et al. Engaging patients to improve quality of care: a systematic review. Implement Sci. 2018;13(1):98. doi: 10.1186/s13012-018-0784-z 30045735 PMC6060529

[10] Chudyk AM, Horrill T, Waldman C, Demczuk L, Shimmin C, Stoddard R, et al. Scoping review of models and frameworks of patient engagement in health services research. BMJ Open. 2022;12(8):e063507. doi: 10.1136/bmjopen-2022-063507 35985787 PMC9396146

[11] International Association for Public Participation. IAP2 Spectrum of Public Participation. https://www.iap2.org/page/pillars. 2024. 2024 October 11.

[12] Heffernan OS, Herzog TM, Schiralli JE, Hawke LD, Chaim G, Henderson JL. Implementation of a youth-adult partnership model in youth mental health systems research: Challenges and successes. Health Expect. 2017;20(6):1183–8. doi: 10.1111/hex.12554 28295940 PMC5689223

[13] Greenhalgh T, Hinton L, Finlay T, et al. Frameworks for supporting patient and public involvement in research: systematic review and co-design pilot. Health Expect. 2019;22:785–801. 31012259 10.1111/hex.12888 PMC6737756

[14] Harrison JD, Auerbach AD, Anderson W, Fagan M, Carnie M, Hanson C, et al. Patient stakeholder engagement in research: A narrative review to describe foundational principles and best practice activities. Health Expect. 2019;22(3):307–16. doi: 10.1111/hex.12873 30761699 PMC6543160

[15] Lea S, Martins A, Morgan S, Cargill J, Taylor RM, Fern LA. Online information and support needs of young people with cancer: a participatory action research study. Adolesc Health Med Ther. 2018;9:121–35. doi: 10.2147/AHMT.S173115 30310338 PMC6167089

[16] Bocquier A, Bruel S, Michel M, Le Duc-Banaszuk A-S, Bonnay S, Branchereau M, et al. Co-development of a school-based and primary care-based multicomponent intervention to improve HPV vaccine coverage amongst French adolescents (the PrevHPV Study). Health Expect. 2023;26(5):1843–53. doi: 10.1111/hex.13778 37312280 PMC10485335

[17] Boivin A, Dumez V, Castonguay G, Berkesse A. The Ecology of Engagement: fostering cooperative efforts in health with patients and communities. Health Expect. 2022;25:2314–27. doi: 10.1111/hex.13571 35923116 PMC9615077

[18] Litterer KP, Cray S, Gonzalez P. Applying coproduction methods to research, clinical care, quality improvement, and education in PHM. Hosp Pediatr. 2024;14:e414–20. 10.1542/hpeds.2023-007448 39175463

[19] Bailey K, Allemang B, Vandermorris A, Munce S, Cleverley K, Chisholm C, et al. Benefits, barriers and recommendations for youth engagement in health research: combining evidence-based and youth perspectives. Res Involv Engagem. 2024;10(1):92. doi: 10.1186/s40900-024-00607-w 39223602 PMC11370084

[20] van Schelven F, Boeije H, Mariën V, Rademakers J. Patient and Public Involvement of young people with a chronic condition in projects in health and social care: A scoping review. Health Expect. 2020;23(4):789–801. doi: 10.1111/hex.13069 32372423 PMC7495073

[21] Allemang B, Cullen O, Schraeder K, Pintson K, Dimitropoulos G. Recommendations for youth engagement in Canadian mental health research in the context of COVID-19. J Can Acad Child Adolesc Psychiatry. 2021;30(2):123–30. 33953764 PMC8056963

[22] Chan M, Scott SD, Campbell A, Elliott SA, Brooks H, Hartling L. Research- and health-related youth advisory groups in Canada: An environmental scan with stakeholder interviews. Health Expect. 2021;24(5):1763–79. doi: 10.1111/hex.13316 34288282 PMC8483214

[23] Oldfield BJ, Harrison MA, Genao I. Patient, family, and community advisory councils in health care and research: a systematic review. J Gen Intern Med. 2019;34:1292–303. 30051331 10.1007/s11606-018-4565-9 PMC6614241

[24] Dardess P, Dokken DL, Unaka NI, Casillas CA, Rouse L, Patel U, et al. Diversity, Equity, and Inclusion in Patient and Family Advisory Councils: Advancing Best Practice in Children’s Hospitals. J Pediatr Health Care. 2024;38(2):184–93. doi: 10.1016/j.pedhc.2023.11.006 38429030

[25] Khalil H, Peters MD, Tricco AC, Pollock D, Alexander L, McInerney P, et al. Conducting high quality scoping reviews-challenges and solutions. J Clin Epidemiol. 2021;130:156–60. doi: 10.1016/j.jclinepi.2020.10.009 33122034

[26] Peters MDJ, Marnie C, Tricco AC, Pollock D, Munn Z, Alexander L, et al. Updated methodological guidance for the conduct of scoping reviews. JBI Evid Synth. 2020;18(10):2119–26. doi: 10.11124/JBIES-20-00167 33038124

[27] Aromataris E, Lockwood C, Porritt K, Pilla B, Jordan Z. JBI Manual for Evidence Synthesis. JBI. 2024. https://synthesismanual.jbi.global

[28] Pollock D, Alexander L, Munn Z, Peters MDJ, Khalil H, Godfrey CM, et al. Moving from consultation to co-creation with knowledge users in scoping reviews: guidance from the JBI Scoping Review Methodology Group. JBI Evid Synth. 2022;20(4):969–79. doi: 10.11124/JBIES-21-00416 35477565

[29] Tricco AC, Lillie E, Zarin W, O’Brien KK, Colquhoun H, Levac D, et al. PRISMA Extension for Scoping Reviews (PRISMA-ScR): Checklist and Explanation. Ann Intern Med. 2018;169(7):467–73. doi: 10.7326/M18-0850 30178033

[30] Boland L, Kothari A, McCutcheon C, Graham ID, Integrated Knowledge Translation Research Network. Building an integrated knowledge translation (IKT) evidence base: colloquium proceedings and research direction. Health Res Policy Syst. 2020;18(1):8. doi: 10.1186/s12961-019-0521-3 31959184 PMC6972018

[31] Warraitch A, Lee M, Bruce D, Curran P, Khraisha Q, Wacker C, et al. An umbrella review of reviews on challenges to meaningful adolescent involvement in health research. Health Expect. 2024;27(1):e13980. doi: 10.1111/hex.13980 39102665 PMC10821743

[32] Fløtten KJØ, Guerreiro AIF, Simonelli I, Solevåg AL, Aujoulat I. Adolescent and young adult patients as co-researchers: A scoping review. Health Expect. 2021;24(4):1044–55. doi: 10.1111/hex.13266 33991369 PMC8369088

[33] Canadian Institutes of Health Research Institute of Genetics. Patient partner compensation guidelines. 2022. https://cihr-irsc.gc.ca/e/documents/ig_compensation-en.pdf

[34] Munce SEP, John T, Luong D, Mooney S, Stromquist L, Chambers K, et al. Establishing Effective Patient Engagement Through a Terms of Reference to Foster Inclusivity and Empowerment in Research: Example From a Healthcare Transition Quality Indicators Project. Health Expect. 2024;27(6):e70113. doi: 10.1111/hex.70113 39610083 PMC11604592

[35] Canadian Institutes of Health Research. Guide to knowledge translation planning at CIHR. 2015. https://cihr-irsc.gc.ca/e/45321.html

[36] Straus SE, Tetroe J, Graham I. Defining knowledge translation. CMAJ. 2009;4(181):165–8. 10.1503/cmaj.081229 PMC2717660 19620273

[37] Toulany A, Gorter JW, Harrison M. A call for action: Recommendations to improve transition to adult care for youth with complex health care needs. Paediatr Child Health. 2022;27(5):297–309. doi: 10.1093/pch/pxac047 36016593 PMC9394635

[38] Canadian Institutes of Health Research. Canada’s Strategy for Patient-Oriented Research. 2011. https://cihr-irsc.gc.ca/e/documents/P-O_Research_Strategy-eng.pdf

[39] Munce SE, Steele Gray C, Pomeroy BC, Bayley M, Kokorelias KM, Luong D, et al. Development of the Preferred Components for Co-Design in Research Guideline and Checklist: Protocol for a Scoping Review and a Modified Delphi Process. JMIR Res Protoc. 2023;12:e50463. doi: 10.2196/50463 37902812 PMC10644195

[40] Park CU, Kim HJ. Measurement of inter-rater reliability in systematic review. Hanyang Med Rev. 2015;35(1):44–9.

[41] Cohen J. A coefficient of agreement for nominal scales. Educ Psychol Meas. 1960;20:37–46.

[42] Munce SEP, Wong E, Luong D, Rao J, Cunningham J, Bailey K, et al. Patient, caregiver and other knowledge user engagement in consensus-building healthcare initiatives: a scoping review protocol. BMJ Open. 2024;14(5):e080822. doi: 10.1136/bmjopen-2023-080822 38719333 PMC11086512

[43] Hsieh H-F, Shannon SE. Three approaches to qualitative content analysis. Qual Health Res. 2005;15(9):1277–88. doi: 10.1177/1049732305276687 16204405

[44] Bergholtz J, Wolf A, Crine V, Cleeve H, Santana M-J, Björkman I. Patient and public involvement in healthcare: a systematic mapping review of systematic reviews - identification of current research and possible directions for future research. BMJ Open. 2024;14(9):e083215. doi: 10.1136/bmjopen-2023-083215 39304210 PMC11418490

[45] Crowther D, McCulloch H, Wong H, Mackay R, Johnson C, Chorney J, et al. Children, young people and parent engagement in health intervention design and implementation: A scoping review. Health Expect. 2023;26(1):1–15. doi: 10.1111/hex.13572 36346148 PMC9854306

[46] Paez A. Gray literature: An important resource in systematic reviews. J Evid Based Med. 2017;10(3):233–40. doi: 10.1111/jebm.12266 28857505

