# Supplementary material for: Commonalities and distinctions of pediatric patient and family engagement in clinical care, education, and research contexts: Protocol for a scoping review
Source: PLoS One. 2025 Aug 8;20(8):e0330104. doi: 10.1371/journal.pone.0330104 (PMC12334048; doi:10.1371/journal.pone.0330104)
Supplement: S1 Appendix — (DOCX) [file pone.0330104.s001.docx]

**S1 Appendix: Literature Search Strategy**

Initial Search Strategy (Run November 21, 2024)

| **#** | **Searches** | **Results** |
| --- | --- | --- |
| 1 | "Delivery of Health Care"/ or Decision making/ or *Health occupations/ed or (((deliver* or system* or provider? or user? or patient?) adj2 (healthcare or "health care")) or "healthcare" or "health care" or decision making or communicat* or health* service? or health* system? or clinical trainee? or "competency-based education" or "educational material? " or "educational intervention?" or "educational resource?" or "medical education").tw,kf. | 1736836 |
| 2 | *Patient Participation/ or (patient-centred research or patient-centered research or "patients as? expert?" or "expert patient?" or ((patient? or youth or consumer? or youth-adult or Y-AP or p?ediatric* or family or families or adolescen* or teen* or child* or young adult?) adj3 (engag* or partner* or involv* or collaborat* or particip*))).tw,kf. | 321072 |
| 3 | *pediatrics/ or Adolescent/ or parents/ or family/ or (adolescen* or teen* or youth* or young person or young people or highschool* or high-school* or patient?).tw,kf. | 10302976 |
| 4 | Community-Based Participatory Research/mt or *Health Services Research/ or (research design* or research method* or research partner* or research personnel* or investigator? or researcher? or Community-Based Participatory Research* or CBPR or PAR or action research* or participative action research* or Consumer-Driven research* or community action-based research* or Research Technique* or research prioriti?ation or Health Care Research* or healthcare research* or cocreat* or codesign* or co-creat* or co-design* or "reciprocal learning" or "bedside teaching" or "bedside learning" or "illness experience" or "lived experience").tw,kf. | 555092 |
| 5 | 1 and 2 and 3 and 4 | 7195 |
| 6 | limit 5 to (english language and yr="2011 -Current") | 6153 |
| 7 | ("36593502" or "31699686" or "33779451" or "35985787" or "29858526" or "28295940" or "34761487" or "29068822" or "30306292" or "35274414").ui. - Key article PMIDs | 10 |
| 8 | 6 and 7 – validation only | 10 |
